# Menstrual waste management practices among female students in Niger delta development commission hostels in educational institutions in Niger delta, Nigeria

**DOI:** 10.1186/s12905-025-03549-x

**Published:** 2025-02-11

**Authors:** Doris Eseoghene Ikogho, Festus Dafe Onoharigho

**Affiliations:** Department of Health and Safety Education, Faculty of Education (DELSU), Delta State University, Abraka, Delta State Nigeria

**Keywords:** Menstrual waste, Management practices, Unsound disposal, NDDC-built hostels, Sustainability

## Abstract

**Background of the study:**

Menstrual waste management encompasses the practices involved in handling menstrual hygiene products from the onset to the conclusion of menstruation. Despite being a crucial public health issue, menstrual waste management remains under-researched, particularly in educational institutions in Nigeria. This study investigates menstrual waste management practices among female students residing in hostels constructed by the Niger Delta Development Commission (NDDC) in the Niger Delta region, focusing on their methods of disposal, associated challenges, and compliance with global standards.

**Methods:**

The study targeted first-year undergraduate female students from three tertiary institutions, with a total population of 825. A systematic sampling technique was used to select a sample of 413 respondents. Data were collected using a structured questionnaire adapted from the World Bank’s Menstrual Health and Hygiene Resource Package (2021) and validated instruments by Adekunle and Ajayi (Am Int J Social Sci Res 4(2):78--87, 2019), with a reliability coefficient of 0.8. Ethical clearance was obtained from Delta State University.

**Result:**

Findings revealed that common menstrual waste materials included cotton wool, pads, and napkins, with many students facing challenges related to affordability and availability of preferred products. Improper disposal practices, such as discarding without wrapping and burying in pits or burning, were prevalent, posing significant health and environmental risks. These unsound practices were exacerbated by the lack of adequate disposal facilities in hostels, limited awareness of proper disposal methods, and non-compliance with global guidelines. Health consequences included an increased risk of infections, while environmental impacts ranged from contamination to prolonged degradation of non-biodegradable materials.

**Conclusion:**

The study highlights the urgent need for infrastructural improvements, targeted education on sustainable menstrual hygiene practices, and stricter adherence to global standards. Recommendations include: University management should jointly implement stringent policies to eliminate unsustainable disposal methods, providing affordable and eco-friendly menstrual products, such as reusable pads, integrating menstrual health education into institutional policies, and enhancing waste management systems. These measures are vital for promoting sustainable menstrual hygiene and aligning with global health and environmental goals.

**Supplementary Information:**

The online version contains supplementary material available at 10.1186/s12905-025-03549-x.

## Introduction

Menstrual waste management involves the practices and activities associated with handling menstrual products from their initial use to disposal. Since 2014, Menstrual Hygiene Day (MHD), celebrated annually on May 28, has sought to challenge taboos, promote proper menstrual hygiene practices, and advocate for their inclusion in policies and programs at all levels [11, 15, 19]. Despite women and girls making up 52% of Nigeria’s population, menstrual waste management remains a critical challenge, compounded by societal stigma and taboos [14, 25].

This issue is particularly pronounced in educational institutions, where large volumes of menstrual waste are generated. Research underscores the need for comprehensive strategies that address both disposal methods and broader hygienic practices. Studies such as those by Adekunle and Ajayi [1] and the World Bank [18] highlight key aspects of menstrual hygiene management (MHM), including designated bins, access to clean water, and education to reduce stigma. Despite progress in some regions, significant gaps persist in addressing these issues within educational settings [19]. Inadequate disposal facilities exacerbate the challenges of managing menstrual waste. A study at the University of Bhutan found that improper disposal of used pads led to environmental pollution [17, 23]. Similarly, in Sub-Saharan Africa, unsanitary disposal practices such as discarding used pads in bathrooms or hiding them under beds create unpleasant odors, increase the risk of skin irritations, and contribute to unhygienic conditions [14, 25, 27]. Exposure to bacteria and pathogens from improperly wrapped used pads further heightens health risks, particularly in environments lacking proper hygiene practices [2, 8, 10]. This underscores the urgent need for improved menstrual hygiene education and infrastructure.

The benefits of sustainable period products are uncountable. Sustainable menstrual products, such as menstrual cups and cloth pads, offer cost-effective and eco-friendly alternatives. These products can be adapted to local customs, reduce reliance on waste management systems, and lower exposure to harmful chemicals, enhancing menstrual hygiene and dignity. They also promote inclusivity and help break cultural taboos surrounding menstruation [23, 25, 26]. Despite these benefits, non-biodegradable sanitary products, used by 36% of females, pose significant health and environmental risks due to their decomposition periods of 500 to 800 years [6, 14, 23]. Advocating for sustainable products and integrating them into policies can reduce waste, improve affordability, and foster better menstrual health [3, 11, 21]. Effective menstrual waste management requires a paradigm shift in perceptions and adherence to global guidelines. For schools, integrating MHM into policies, addressing resource gaps, and fostering supportive environments are essential. Such efforts align with the Sustainable Development Goals (SDGs) 4 and 5, focusing on inclusive education and gender equality [4, 5, 12, 22]. Socio-cultural norms and the pandemic are also evident. These two duo significantly influence menstrual health practices. In India, menstrual health management is deeply influenced by socio-cultural norms. Menstruating women in rural areas often face restrictions, such as being excluded from kitchens and religious practices, driven by beliefs of impurity. Orthodox families may impose physical isolation during menstruation, while some communities celebrate menarche with ceremonies like the “Ritushuddhi” ritual, marking a girl’s transition to womanhood [7, 13, 28]. This duality highlights empowerment alongside stigma. Mudi et al. [7, 10, 24] emphasized the need to address these cultural barriers through culturally sensitive health education, integrating menstrual health into public health policies to support rural and tribal populations.

Recent reports from 2024 show shifts in societal attitudes, including declining acceptance of restrictive practices and increased efforts to normalize menstruation through comprehensive health education and workplace policies [7, 12, 30]. The COVID-19 pandemic further disrupted menstrual waste management practices globally, including in Nigeria and India. Service interruptions and economic constraints during the pandemic forced many to rely on alternatives like cotton wool and reusable cloths, contributing to unsafe disposal practices. These unsanitary methods exacerbated health risks, increased exposure to pathogens, and heightened environmental pollution [4, 7, 12, 16].

Global guidelines and localized efforts from the World Bank’s Menstrual Health and Hygiene Resource Package [29] and studies by Adekunle and Ajayi [1, 20, 22] emphasized access to clean water, designated bins, and education on proper disposal methods. Regular training and community engagement are important ingredients in ensuring compliance and promote menstrual dignity. Addressing cultural and socio-demographic factors in shaping menstrual health practices remains a cornerstone of effective policy design [4, 5, 9].

In the Niger Delta region, the Niger Delta Development Commission (NDDC) has sought to address these challenges by constructing hostels in federal tertiary institutions. This study fills a critical research gap by focusing on menstrual waste management practices in NDDC-built hostels. Unlike prior studies that focused on broader community issues, this research examines context-specific challenges, such as infrastructural limitations and compliance with global guidelines. By providing insights into disposal behaviors, cultural barriers, and health risks, this study highlighted the need for localized policies and improved infrastructure. Addressing these challenges is vital to fostering sustainable menstrual hygiene management in Nigerian universities. This study therefore focuses on examining menstrual waste management practices specifically within NDDC-built hostels in the Niger Delta region.

## Statement of problem

Menstrual waste management is a critical yet neglected issue in Nigeria, where young females make up 52% of the population. In educational institutions, particularly in NDDC-built hostels, improper disposal methods, such as flushing used pads down toilets or discarding them into water bodies, lead to blockages, the spread of germs, and pose severe health risks, including the potential survival of viruses like hepatitis and HIV. These issues are worsened by inadequate sanitation facilities and lack of clean water. Despite the significance of the problem, there is limited research on menstrual waste management in university settings, particularly in the Niger Delta region. Current practices often fail to comply with global hygiene standards, and insufficient education on menstrual hygiene exacerbates improper disposal behaviors. This study aims to explore menstrual waste management practices among female students in NDDC hostels, addressing gaps in infrastructure, awareness, and policy, while advocating for sustainable solutions to improve health and sanitation.

### Objective of the study

The main objective of the study is to explore menstrual waste management practices among female students at NDDC hostels in the Niger Delta. Specific objectives include:

1. Identifying materials used for menstrual flow by females in NDDC hostels.
2. Determining the challenges faced by students in managing menstrual waste and evaluating the available infrastructure/methods for disposal.
3. Identifying the health and environmental impacts of inadequate menstrual waste management experienced by female students.
4. Proposing solutions and interventions to improve menstrual waste management in NDDC-built hostels, contributing to a healthier and sustainable environment.

## Research questions

The research questions for this study are:

1. What materials are currently used by female students for managing menstrual waste?
2. What methods are used to dispose of menstrual waste at NDDC hostels?
3. What health risks are associated with inadequate menstrual waste management in NDDC-built hostels?
4. How do global guidelines align with menstrual waste management practices in NDDC-built hostels?

## Materials and methods

This study utilized a survey research design based on the study’s objectives. The population comprised all female first year students residing in NDDC hostels across three educational institutions in the Niger Delta region, totaling 825 students for the 2020/2021 academic session. The age of the respondents ranged from 17 to 25 years, with a mean age of 21 years. A manageable sample size of 413 respondents was selected using systematic sampling, from a population of 825 for the study, by applying the following statistical method,: With a 95% confidence level and a 5% margin of error, the sample size calculation: was done by using the finite population formula:

$$n=\frac{N\cdot Z^{2}\cdot p \cdot (1-p)}{E^{2}\cdot (N-1)+Z^{2}\cdot p \cdot (1-p)}$$

where N = 825 N = 825 N = 825, Z = 1.96Z = 1.96Z = 1.96,p = 0.5p = 0.5p = 0.5, and E = 0.05E = 0.05E = 0.05, the calculation yielded a sample size of approximately 413.

The data collection instrument was a questionnaire adapted from the World Bank’s Menstrual Health and Hygiene Resource Package (2021) and a tool by Adekunle & Ajayi (2019) [1, 18]. The questionnaire was validated by experts in Health and Safety Education, and its reliability was confirmed through a test-retest procedure with a coefficient of 0.8, allowing for a 5% margin of error. To assess health risks, the study used a survey to measure the frequency of specific health issues caused by improper menstrual waste management. Key health indicators included dermatitis, irritation, rash, unpleasant odors, disruption of vaginal pH balance, and environmental contamination. Responses were analyzed using mean scores, representing the average frequency and severity of these health issues on a scale of 1 to 3. Dermatitis had a mean score of 2.319, indicating moderate frequency and severity, while irritation had a mean score of 2.709, making it the most prevalent issue, experienced by over 90% of respondents. Rash, with a mean score of 2.569, was reported by 85.67% of respondents. In contrast, bad odor had a mean score of 1.314, suggesting it was less commonly experienced. These mean scores reflect the overall severity of the health problems and help identify which risks need to be prioritized for intervention.

The study’s data collection occurred over a two-month period, from March to April 2022, during the second semester of 2022-23 academic session. Ethical approval was obtained from the Ethical Committee of Delta State University (Approval No: RBC/FBMC/DELSU/24/465) to ensure adherence to ethical standards. The collected data were analyzed and presented in the following tables below (see Tables 1, 2, 3, 4 and 5).

## Results

### Research question 1

What materials are used for menstrual flow by females in NDDC hostels?

**Table 1:** Materials used for Menstrual Flow by females of NDDC Hostels

Materials Used	Yes (%)	No (%)
Cotton Wools	60.2	39.5
Regular Pads	72.3	27.4
Old Clothes	12.8	86.7
Tampons	4.8	95.2
Sanitary Napkins	97.6	2.4

Data analyses showed that the most commonly used materials are sanitary napkins (97.6%), regular pads (72.3%), and cotton wools (60.2%). Old clothes (12.8%) and tampons (4.8%) are less frequently used. The study identified several products used by the female students, with varying degrees of popularity, reflecting both cultural and accessibility challenges.

**Table 2:** Products used for with solo percentages

Products	Used (%)
Sanitary Napkins	97.6%
Regular Pads	72.3%
Cotton Wool	60.2%
Old Clothes	12.8%
Tampons	4.8%

Products used with solo percentages are as follows:

Sanitary Napkins (97.6%): Almost universally used, sanitary napkins dominate as the primary product, showing they are accessible and preferred due to their absorbency and comfort.

Regular Pads (72.3%): Widely used, these are the second-most popular, offering a balance between affordability and availability.

Cotton Wool (60.2%): More than half of the students used cotton wool, likely due to its lower cost and easy availability, though it’s less hygienic compared to napkins and pads.

Old Clothes (12.8%): While not commonly used, old clothes reflect a lack of access to better products among a minority of students. Tampons (4.8%): Tampons have the lowest usage, possibly due to cultural stigma or lack of availability in the region. The results show several intersections: Many students use a combination of products, possibly switching between sanitary napkins and regular pads depending on availability or financial constraints. Cotton wool use might overlap with old clothes, as they are both low-cost alternatives, suggesting economic factors influence these choices. The low usage of tampons may intersect with the high use of other products, as tampons could be reserved for special occasions or physical activities that require discretion.

### Research question 2

How is menstrual waste managed at the NDDC-built hostels?

**Table 3:** Methods used to Manage Menstrual Waste at NDDC Hostels

Methods to Manage Menstrual Waste	Yes (%)	No (%)
Dispose in Designated Bin	82.3	17.7
Flushed in Toilet	27.8	72.2
Burying in Pits	96.4	3.6
Burning	61.7	38.0
Wrap and Dispose in General Waste Bin	3.1	96.9
Socially Unacceptable Manner	75.3	24.7

The data analyses revealed a concerning reliance on unsound menstrual waste management practices among female students residing in NDDC-built hostels. While 82.3% of respondents reported disposing of menstrual waste in designated bins, the prevalence of unsound methods remains alarmingly high. For instance, 96.4% of students bury menstrual waste in pits, and 61.7% resort to burning. Additionally, 75.3% dispose of menstrual waste in socially unacceptable ways, including indiscriminate tossing, while only 3.1% wrap and dispose of waste in general bins. The prevalence of these unsound practices has significant health and environmental repercussions. Highlighting the need for stricter policies, enforcement, and education.

### Research question 3

What are the health risks associated with menstrual waste management at NDDC-built hostels?

**Table 4:** Health Risks Associated with Menstrual Waste Management at NDDC Hostels

Health Problems Experienced	Mean	Std Deviation	Mean Percent (%)
Dermatitis	2.3196	0.7565	77.33
Irritation	2.7094	0511	90.33
Rash	2.5690	0.5645	85.67
Bad Odour	1.3148	0.5376	43.67
Alteration of Vaginal pH Secretion	2.2615	0.8240	75.33
Environmental Contamination	2.6465	0.6239	88.33

mean scores = quantify the severity, The total score = the aggregated average responses

Data analyses captured health issues linked to menstrual waste practices. Notable findings include:

90.33% of respondents reported irritation, 85.67% reported rashes. Dermatitis affected 77.33%, while bad odor (43.67%) and altered vaginal pH (75.33%) were common complaints. Highlighting the broader impact of improper waste disposal methods. These findings underscore the necessity of hygienic disposal systems and education.

### Research question 4

What global guidelines align with menstrual waste management in NDDC-built hostels?

**Table 5:** Global guidelines on management of Menstrual Waste at NDDC Hostels

Global Guidelines	Always (%)	Sometimes (%)	Never (%)
Access to Constant Clean Water Supply	49.6	32.7	17.7
Use of Proper Designated Bins	75.8	19.4	4.8
Education on Proper Disposal	68.2	24.5	7.3
Regular Training on Menstrual Hygiene	62.0	31.8	6.2

The data analyses showed that while most institutions have designated bins and provide education on disposal (75.8% and 68.2%, respectively), access to constant clean water supply is less frequently addressed (49.6%). Implementing global best practices can help mitigate health risks and promote sustainable waste management systems.

### Discussion of findings

The findings reveal critical gaps in menstrual waste management practices within NDDC-built hostels, reinforcing patterns observed in existing literature. The widespread use of disposable products, particularly sanitary napkins (97.6%) and regular pads (72.3%), aligns with global trends Adekunle and Ajayi [1]; World Bank [29]. However, the high prevalence of alternatives like cotton wool (60.2%) and old clothes (12.8%) highlighted economic disparities and restricted access to preferred products, as seen in studies on low-resource settings (Mudi et al., [10]. Despite the availability of disposal options such as designated bins (82.3%), unsustainable methods like burying in pits (96.4%) and burning (61.7%) remain dominant. These practices, while convenient, pose significant environmental and public health risks, resonating with findings in Sub-Saharan Africa (World Bank [29]. The socially unacceptable nature of these practices, reported by 75.3% of respondents, reflects the stigma surrounding menstruation and the lack of adequate waste management education. This aligns with studies by Mudi et al. [10], as seen in areas with high literacy.

The study further highlights the adverse health implications associated with poor menstrual waste management. Irritation (90.33%), rashes (85.67%), and dermatitis (77.33%) were the most frequently reported conditions, aligning with established correlations between inadequate hygiene practices and dermatological or infectious health risks [1]. The alteration of vaginal pH (75.33%) and unpleasant odors (43.67%) add to these concerns, emphasizing the need for clean disposal environments and adherence to global hygiene standards [4, 5, 9, 29].

The reported lack of access to basic amenities, such as consistent water supply (49.6%), and inadequate regular training on menstrual hygiene (62.0%) further hinders the adoption of safe practices. These findings echo global concerns, such as those outlined in the World Bank’s Menstrual Health and Hygiene Resource Package, where infrastructural deficits and insufficient education remain significant barriers to progress [29].

Cultural influences, including menstrual stigma and taboos, exacerbate these challenges [7, 10, 12]. Practices in the Niger Delta reflect findings in India, where cultural restrictions limit discourse and contribute to unsafe disposal methods [7, 10, 12]. Addressing these deeply ingrained norms requires culturally sensitive interventions, such as targeted educational campaigns and community engagement. To advance menstrual waste management, policies must prioritize access to affordable, sustainable menstrual products, adequate water supply, and effective waste disposal systems. Ending unsound practices such as burying in pits and burning demands the integration of environmental sustainability in public health policies [14, 27]. Additionally, enforcing menstrual hygiene education within university curricula will empower young women with knowledge and practical skills, fostering behavioral change. These findings emphasize the urgent need for multi-sectoral collaboration to implement policies that align with the Sustainable Development Goals, particularly SDGs 3 (Good Health), 4 (Quality Education), 5 (Gender Equality), and 6 (Clean Water and Sanitation). By addressing systemic barriers and promoting sustainable practices, stakeholders can significantly enhance menstrual health management and contribute to achieving equity and dignity for all women.

### Alignment with global guidelines

While the study identified pockets of adherence to global MHM guidelines, such as the use of designated bins, significant gaps remain in areas like education and regular training. These shortcomings highlight the need for stronger alignment with global frameworks such as the Menstrual Health and Hygiene Resource Package as observed by World Bank [29]. Institutional adherence to these guidelines can drive sustainable improvements in waste management practices and reduce environmental and health risks.

### Comparative insights from the India study

1. Environmental Impact: Both studies emphasize the unsustainable environmental burden of menstrual waste. India’s alarming statistic of 12 billion non-biodegradable pads discarded annually underscores the urgency of adopting reusable alternatives, which is echoed in the NDDC context.
2. Access to Quality Products: Both highlight limited access to quality menstrual products for marginalized populations, with issues of durability and availability being critical in both contexts.
3. Education: Both studies emphasize menstrual education to reduce stigma and improve hygiene, underscoring the need for comprehensive programs.

### Divergences

1. Inclusivity: The India study includes transgender and non-binary individuals in menstrual health discussions, whereas the NDDC study focuses solely on female students.
2. Policy Approach: India advocates for centralized, nationally coordinated policies, while the NDDC study emphasizes localized, hostel-specific solutions.
3. Target Groups: India extends support to out-of-school individuals, while the NDDC study focuses on students in hostels.

### Implications

1. Public Health: Improved menstrual hygiene management can reduce infections and associated health burdens, enhancing the quality of life for students.
2. Environmental Sustainability: Addressing improper disposal practices through eco-friendly alternatives mitigates environmental degradation and aligns with global sustainability goals.
3. Policy Development: The findings underscore the need for localized solutions supported by broader national frameworks, fostering effective menstrual waste management in university environments.
4. Educational Interventions: Comprehensive menstrual health education is critical in addressing stigma, improving hygiene behaviors, and promoting adherence to global guidelines.

### Broader public health implications

1. Increased Disease Burden; unsanitary practices stemming from inadequate resources and education can lead to higher incidences of infections and communicable diseases in both individuals and communities.
2. Environmental Pollution and Indirect Health Risks: improper disposal methods, such as burning or open dumping, contribute to air, soil, and water pollution, exacerbating health risks like respiratory problems and exposure to toxic substances.

### Barriers to gender equity in health

1. Menstruators who face challenges in managing their hygiene due to inadequate access to water or educational gaps may experience stigma, reduced school attendance, and overall poorer health outcomes.
2. Strain on Healthcare Systems: the cumulative health impacts of inadequate menstrual waste management can increase healthcare burdens, diverting resources to address preventable conditions.

### Recommendations

#### Elimination of unsound disposal practices

Environmental health agencies, university administrators, and hostel management should jointly implement stringent policies to eliminate unsustainable disposal methods such as open dumping, pit burying, and burning. This requires the installation of environmentally compliant systems, such as regulated incinerators and waste processing units, to ensure safe and sustainable disposal practices while minimizing environmental pollution.

#### Infrastructure development for sanitation

University administrations and funding bodies should allocate resources for upgrading hostel sanitation facilities, ensuring uninterrupted access to clean water and adequate disposal options. National and regional policymakers must prioritize investments in these facilities to align with global hygiene and environmental standards, addressing existing infrastructure disparities.

#### Awareness and education programs

Comprehensive menstrual hygiene education programs should be developed and implemented by health educators and public health specialists. These programs should aim to inform students about proper disposal methods, environmental impacts, and associated health risks. School administrators and policymakers must integrate these educational initiatives into the institutional curriculum to dispel myths and reduce stigma surrounding menstruation and support awareness campaigns.

#### Promotion of sustainable menstrual products

University policymakers, government and NGOs should promote the adoption of reusable and eco-friendly menstrual products such as menstrual cups and cloth pads. Strategies should include subsidies for sustainable products, awareness campaigns, and partnerships with manufacturers to enhance affordability and accessibility, particularly in under-resourced settings.

#### Policy formulation and enforcement

University administrations, in partnership with policymakers, should establish clear and enforceable guidelines for menstrual waste management in hostels and other educational facilities. These guidelines must align with global best practices and include mechanisms for regular monitoring and evaluation by environmental health officers.

## Limitations

1. The study focused exclusively on female students in NDDC hostels, which may not fully represent menstrual waste management practices in other university hostels or broader communities in Nigeria.
2. The reliance on self-reported data from students may introduce bias, as respondents might underreport improper practices or be unaware of proper disposal methods.
3. The study was conducted in a specific region (Niger Delta), and the findings may not be generalizable to other regions in Nigeria with different cultural or infrastructural contexts.
4. The study was done across sections, providing a snapshot of current practices. Longitudinal studies could provide deeper insights into how practices evolve over time and in response to interventions.

## Electronic supplementary material

Below is the link to the electronic supplementary material.

Supplementary Material 1

Supplementary Material 2

## Data Availability

The raw data generated for the study are available within the manuscript.
