# Supplementary material for: Menstrual waste management practices among female students in Niger delta development commission hostels in educational institutions in Niger delta, Nigeria
Source: BMC Womens Health. 2025 Feb 11;25:60. doi: 10.1186/s12905-025-03549-x (PMC11816509; doi:10.1186/s12905-025-03549-x)
Supplement: Supplementary file 1 — Supplementary Material 1 [file 12905_2025_3549_MOESM1_ESM.docx]

RAW DATA USED FOR THE

Materials used for menstrual flow by females of NDDC hostel

| Materials used | Yes | No |
| --- | --- | --- |
| 1. Cotton wools | 250 | 163 |
| 2. Regular pads | 300 | 113 |
| 3. Old clothes | 53 | 360 |
| 4. Tampons | 20 | 393 |
| 5. Sanitary napkins | 403 | 10 |

Methods used to manage menstrual waste at NDDC built hostels

| Methods to manage menstrual waste at NDDC hostels | Yes | No |
| --- | --- | --- |
| 1. Dispose in designated bin | 340 | 73 |
| 2. Flushed in toilet | 115 | 298 |
| 3. Burying in pits | 398 | 15 |
| 4. Burning | 256 | 157 |
| 5. Wrap and dispose in general waste bin | 13 | 400 |
| 6. socially unacceptable manner | 311 | 102 |

Health risks associated with management of menstrual waste at the NDDC built hostels

| Heath Risks | Yes | No |
| --- | --- | --- |
| 1. Dermatitis | 320 | 93 |
| 2. Irritation | 373 | 40 |
| 3. Rash | 354 | 59 |
| 4. Bad odour | 180 | 233 |
| 5. Alteration of vaginal ph secretion of | 312 | 101 |
| 6.Environmental contamination | 365 | 48 |
