# Supplementary material for: Menstrual waste management practices among female students in Niger delta development commission hostels in educational institutions in Niger delta, Nigeria
Source: BMC Womens Health. 2025 Feb 11;25:60. doi: 10.1186/s12905-025-03549-x (PMC11816509; doi:10.1186/s12905-025-03549-x)
Supplement: Supplementary file 2 — Supplementary Material 2 [file 12905_2025_3549_MOESM2_ESM.docx]

**RESPONSE TO REVISION**

**SUPPLEMENTARY FILE ON:**

Menstrual Waste Management Practices Among Female Students In Niger Delta Development Commission Hostels In Tertiary Institutions In Niger Delta, Nigeria.

**Response to Revision**

1. The instrument used for data collection was a questionnaire adapted from the tools used by World Bank on Menstrual Health and Hygiene Resource Package in 2021.. Retrieved from

[World Bank, https://documents1.worldbank.org › curated › pdf](  World Bank, https://documents1.worldbank.org › curated › pdf)

and those used by Adekunle, O. & Ajayi, E. in 2019 with a -DOi - 10.46281/aijssr.v4i2.350. Retrieved from https://www.researchgate.net>341.... [13,16].

**Data collection tool**

| Part 1: Socio-demographic related questions | | | |
| --- | --- | --- | --- |
| **S.No** | **Questions** | **Possible answer** | **Remark** |
|  | How old are you now? | I am _________years old |  |
|  | School type? | 1. Public 2. Private |  |
|  | Grade level? | 1. Grade 9th, 2. Grade 10th, 3. Grade 11th, 4. Grade 12^th^ |  |
|  | How old were you at your menarche? | I was ------------years old. |  |
|  | Residence? | 1. Urban 2. Rural |  |
|  | Religion? | 1. Orthodox 2. Muslim 3. Protestant 4. Others; specify |  |
|  | Ethnicity? | 1. Amhara 2. Oromo 3. Tigre 4. Other; specify--- |  |
|  | What is your father’s educational status? | 1. Can’t read and write 2. Primary 3. High school 4. Secondary and preparatory 5. College diploma 6. University degree |  |
|  | What is your mother’s educational status? | 1. Can’t read and write 2. Primary 3. High school 4. Secondary and preparatory 5. College diploma 6. University degree |  |
|  | What is the occupational status of your father? | 1. Government Employee 2. Private Employee 3. Self-Employee 4. Others; specify----------- |  |
|  | What is the occupational status of your mother? | 1. Government Employee  2. Private Employee  3. Self-Employee  4. Others; specify------------- |  |
|  | How much does your family earn per month on average? | 1. <1000 2. 1000 -2000 3. >2001 4. don’t know |  |
|  | Do your parents provide permanent pocket money regularly? | 1. Yes  2. No |  |
| **Part 2: - Knowledge and awareness regarding menstruation** | | | |
|  | At what age, a girl does commence her menarche? | At ----------years old |  |
|  | What is the cause of menstruation? | - 1. Physiological process.   2. Is caused by sin.   3. Is the curse of God.   4. Is caused by a disease.   5. I don't know |  |
|  | From which organ does the menstrual blood come? | 1. Vagina  2. Urinary bladder  3. Uterus  4. I don't know |  |
|  | What absorbent should be ideally used during menstruation? | 1. Disposable sanitary pad  2. Reusable and washable cloth pads.  3. Rag or pieces of cloth  4. Other, specify-------- |  |
|  | How long is the normal menstrual bleeding duration? | 1. <2 Days.  2. 2-7 Days  3. >7 Days  4. Don’t know |  |
|  | What is the normal duration of the menstrual cycle? | 1. <20 Days  2. 20-35 Days  3. >35 Days  4. Don’t know |  |
|  | Can a girl go to school during menstruation? | 1. Yes 2. No |  |
|  | Is menstruation a secret issue? | 1. Yes 2. No |  |
|  | If your answer is yes for question no 022, why? | 1. Deep-rooted culture of the society. 2. Believes and customs of the society 3. Both 4. Others; specify----------- |  |
| **Part 3: -Menstrual hygiene management practice and related questions.** | | | |
|  | Do you use sanitary material(s) during menstruation? | 1. Yes  2. No |  |
|  | If your answer is Yes for Q no 024, what sanitary material do you use during menstruation? | 1. Disposable sanitary pads.  2. Disposable piece of rags.  3. Reusable sanitary pads  4. Paper/toilet paper.  5. Underwear.  6. Others; specify-------- |  |
|  | If your answer is No for Q no 025, why? | 1. Lack of knowledge  2. High cost  3. Unavailability  4. Shyness  5. Others; specify----- |  |
|  | Do you wash your genitalia during menstruation? | 1. Yes  2. No |  |
|  | If your answer to question no 027 is yes what medium do you use for your genital cleaning purpose? | 1. Only Water.  2. Soap and water.  3. Others; specify-------- |  |
|  | If your answer for question no 027 is yes how often do you wash your genitalia per day? | 1.Twice  2. Thrice  3. >=Four times |  |
|  | Do you take bath during menstruation (exceptional from the usual)? | 1. Yes  2. No |  |
|  | If your answer to question no 030 is yes, how often do you take bath during menstruation per day? | - 1. <= Two times in a day  1. 2. > Two times in a day |  |
|  | Do you change your sanitary material(s) during menstruation at school? | 1. Yes 2. No |  |
|  | If your answer for question no 032 is yes how often do you change your sanitary material (s) during menstruation per day? | 1. 1 to 6 times  2. ≥ 7 times |  |
|  | How do you dispose of menstrual materials after use? | 1. Open field  2. Latrine  3. Wrap in paper and put in the bin  4. Others; specify ------------ |  |
|  | Where do you store your new and/or reusable absorbent(s)? | 1. Drawers  2. Dress cabinet  3. Bathrooms.  4. Store with a routine cloth.  5. Don’t store  6. Others; specify-- |  |
|  | Where do you put/keep your reusable sanitary pads after washing for drying? | 1. In the shade outside  2. In the shade inside  3. In the sunlight inside  4. In the sunlight outside  5. Hidden under other clothes  6. Hidden elsewhere  7. Other; specify---- |  |
|  | Have you heard about menstruation before menarche? | 1. Yes  2. No |  |
|  | If your answer is ‘Yes’ for question no 037, where did you get the information about menstruation before menarche? | 1. Mother. 2. Teacher. 3. Health personnel. 4. TV 5. others; specify------------- |  |
|  | Do you freely discuss menstruation issues with your parents? | 1. Yes  2. No |  |
|  | If your answer for question no 38 is “Yes”, in what topics/issues? | 1. About menstrual hygiene management. 2. About methods of how to use sanitary pads 3. All |  |
|  | If your answer for question no 039 is “No”, why? | 1. Because of shamefulness  2. Not habitual.  3. Privacy.  4. All |  |
|  | Do you know sanitary pads in the market? | 1. Yes 2. No |  |
|  | Does the school have a water source? | 1. Yes 2. No |  |
|  | Does the school have a toilet facility? | 1.Yes 2. No |  |
|  | Are females' and males’ toilets in the opposite directions? | 1. Yes 2. No |  |
|  | Are females’ toilets kept locked inside? | 1. Yes 2. No |  |
